# A Systematic Review and Network Meta-Analysis of Biologic Agents in the First Line Setting for Advanced Colorectal Cancer

**DOI:** 10.1371/journal.pone.0140187

**Published:** 2015-10-16

**Authors:** Alexander Kumachev, Marie Yan, Scott Berry, Yoo-Joung Ko, Maria C. R. Martinez, Keya Shah, Kelvin K. W. Chan

**Affiliations:** 1 Faculty of Medicine, University of Toronto, Toronto Canada; 2 Sunnybrook Odette Cancer Centre, University of Toronto, Toronto, Canada; 3 Faculty of Medicine, Queens University, Kingston, Canada; 4 Division of Biostatistics, Dalla Lana School of Public Health, University of Toronto, Toronto, Canada; University Campus Bio-Medico, ITALY

## Abstract

**Background:**

Epithelial growth factor receptor inhibitors (EGFRis) and bevacizumab (BEV) are used in combination with chemotherapy for the treatment of metastatic colorectal cancer (mCRC). However, few randomized controlled trials (RCTs) have directly compared their relative efficacy on progression-free survival (PFS) and overall survival (OS).

**Methods:**

We conducted a systematic review of first-line RCTs comparing (1) EGFRis vs. BEV, with chemotherapy in both arms (2) EGFRis + chemotherapy vs. chemotherapy alone, or (3) BEV + chemotherapy vs. chemotherapy alone, using Cochrane methodology. Data on and PFS and OS were extracted using the Parmar method. Pairwise meta-analyses and Bayesian network meta-analyses (NMA) were conducted to estimate the direct, indirect and combined PFS and OS hazard ratios (HRs) comparing EGFRis to BEV.

**Results:**

Seventeen RCTs contained extractable data for quantitative analysis. Combining direct and indirect data using an NMA did not show a statistical difference between EGFRis versus BEV (PFS HR = 1.11 (95% CR: 0.92–1.36) and OS HR = 0.91 (95% CR: 0.75–1.09)). Direct meta-analysis (3 RCTs), indirect (14 RCTs) and combined (17 RCTs) NMA of PFS HRs were concordant and did not show a difference between EGFRis and BEV. Meta-analysis of OS using direct evidence, largely influenced by one trial, showed an improvement with EGFRis therapy (HR = 0.79 (95% CR: 0.65–0.98)), while indirect and combined NMA of OS did not show a difference between EGFRis and BEV Successive inclusions of trials over time in the combined NMA did not show superiority of EGFRis over BEV.

**Conclusions:**

Our findings did not support OS or PFS benefits of EGFRis over BEV in first-line mCRC.

## Introduction

Colorectal cancer (CRC) is among the most common forms of cancer worldwide [1]. CRC has the third highest cancer incidence and rate of death in the USA for men and women, even though national incidence and mortality rates have been steadily declining in the past decades [2]. In addition, recent studies [3] report increased rates of CRC in economically transitioning countries around the world. Among patients already diagnosed with colorectal cancer, approximately one-fifth are diagnosed with synchronous metastasis, and half of the remaining patients will develop subsequent metastases [4,5]. For most patients with metastatic CRC (mCRC), treatment is palliative rather than curative [6], with an overall 5-year survival at approximately 10% [2].

Several cytotoxic agents have demonstrated efficacy in the treatment of mCRC, including 5-fluorouracil (5-FU), folinic acid, irinotecan, oxaliplatin and capecitabine. These drugs are commonly combined in FOLFOX (folinic acid, fluorouracil and oxaliplatin), FOLFIRI (folinic acid, fluorouracil and irinotecan), or XELOX (capecetabine and oxaliplatin) regimens, and can be further combined with monoclonal antibody therapy [7, 8, 9, 10, 11, 12]. Two antibody classes that have been shown to improve treatment outcomes for mCRC when combined with chemotherapy include antibodies targeting the vascular endothelial growth factor (VEGF), such as bevacizumab (BEV), and targeting the epidermal growth factor receptor (EGFR), including cetuximab and panitumumab [10,13]. The efficacy of EGFR inhibitors (EGFRis) has been found to vary with patient populations. Several studies [14,15] have shown that benefits of EGFRis are limited to patients whose tumours are K-RAS wild-type.

To date, three randomized studies [16, 17, 18] have been reported (one phase II and two phase III trials), that directly compared the efficacies of BEV with EGFRis when combined with a chemotherapy regimen as first-line treatment in mCRC. The results of the phase III FIRE-3 study suggested cetuximab did not improve progression-free survival (PFS), but significantly improved response rate (RR) and overall survival (OS) in patients with *K-RAS* Exon 2 wild-type advanced colorectal cancer [19]. The phase II PEAK trial, similarly showed improvement in the OS without improvement in the PFS among the *K-RAS* wild-type group [18]. In contrast, the larger phase III trial (CALGB 80405)—and only one powered for OS—showed no improvement in OS or PFS with the use of cetuximab when compared with bevacizumab in patients with *K-RAS* wild-type [20]. Several studies [19,21] have shown that the addition of cetuximab or panitumumab to bevacizumab (i.e. dual biologic therapies) in patients receiving chemotherapy for CRC increased the rate of adverse events, with mixed or worse therapeutic outcomes. Choosing the most effective antibody therapy to combine with first-line chemotherapy remains an important consideration, but the data informing this choice is conflicting.

Traditional meta-analyses are helpful in providing a direct comparison between trials with the same intervention and comparator. However, in settings when few or no direct comparisons of treatments exist, an indirect comparison approach [22] allows for the comparison of treatments between groups from different trials, if the studies have a common treatment parameter [23,24]. The use of a network meta-analysis (NMA) by combining direct evidence with indirect evidence can often increase the precision of the comparison [25]. NMAs have been recently conducted in a number of oncology settings including metastatic breast cancer [26], metastatic pancreatic cancer [27], adjuvant treatment for pancreatic cancer [28] and Hodgkins lymphoma [29] to simultaneously examine the relative efficacy of multiple treatments by synthesizing both direct and indirect evidence.

In this study, we carried out a systematic review of all randomized controlled trials (RCTs) that compared systemic chemotherapy regimens with and without bevacizumab, cetuximab or panitumumab for the first-line treatment of mCRC and conducted both direct meta-analysis, and indirect and combined NMAs to assess the impact of these agents on PFS and OS.

## Methods

### Search strategy and selection criteria

We performed a systematic review to identify studies which examined the survival outcomes of mCRC patients. Under consideration for inclusion were all RCTs which compared chemotherapy treatment alone to either i) chemotherapy treatment in combination with BEV or ii) chemotherapy in combination with EGFRis. RCTs that directly compared chemotherapy treatment with EGFRis against chemotherapy combined with BEV were also considered. Studies were included regardless of the chemotherapy backbone as long as the backbone was identical in both the treatment and control arms. Studies were included only if the patients were being treated with first-line therapy for the treatment of mCRC. Those studies with more than two treatment arms were included if one of more of the arms included an eligible comparison; only arms addressing eligible comparisons were included in the analysis. For EGFRis trials, only data from *K-RAS* participants with wild-type *K-RAS* Exon 2 tumors was included.

We excluded non-randomized trials, as well as trials involving non-metastatic colorectal cancer patients. Trials that included radiotherapy, hormonal therapy, gene therapy or other immunologic therapy in one of the arms were excluded. Studies with a comparison of chemotherapy and a *VEGFi* or an EGFRi against no treatment (best supportive care), or against a *VEGFi* or EGFRi alone were excluded. When several reports discussed the same trial, the report with the most recent data was included.

We searched Medline, Embase, and the Central Registry of Controlled Trials of the Cochrane Library. All databases were updated through to the second week of September, 2014. We did not place any language restrictions on the search. The complete search strategy employed has been provided (S1 Text). Our review has been reported using the PRISMA reporting guidelines (S1 PRISMA Checklist).

### Data extraction

Data was extracted by two independent reviewers with discrepancies between the reviewers discussed prior to selecting trials for inclusion in the systematic review and prior to inclusion in the meta-analysis. Unresolved discrepencies were reviewed by a third reviewer. We recorded primary author, trial ID, treatment comparison, primary and secondary outcomes studied, location of the trial, recruitment period, number of patients randomized and evaluated in each treatment arm, age, gender, and K-RAS status.

The studies were organized into three treatment groups; chemotherapy with BEV against chemotherapy alone, chemotherapy with an EGFRi against chemotherapy alone, and chemotherapy with BEV against chemotherapy with an EGFRi. For our analysis, studies examining cetuximab or panitumumab were grouped together as EGFRi. Studies with different chemotherapy backbones, methods of chemotherapy administration, or methods of antibody therapy administration were grouped according to the type of antibody therapy used. In trials involving a comparison with an EGFRi, only data pertaining to wild-type *K-RAS* Exon 2 patients was extracted.

For each trial, we recorded PFS and/or OS of the treatment and control arms, the hazard ratio (HR), log-rank p-value, and confidence intervals when available. In studies where the HR, p-value, or confidence intervals were not provided, it was calculated from the log-rank p value, the number of events in each arm and the number of randomized patients in each arm using the Parmar method [30].

### Statistical analysis

Pairwise meta-analyses were conducted to examine treatment regimens that were directly compared in the studies; chemotherapy vs chemotherapy with EGFRis, chemotherapy vs chemotherapy with BEV, and EGFRis vs BEV with chemotherapy in both arms. The results were combined into forest plots using Review Manager, version 5.2, using the random-effects model. To assess the comparability of included studies, between-study heterogeneity was estimated and reported using the I^2^ statistic; the value of I^2^ lies between 0% and 100%, where 0% indicates no observed heterogeneity and larger values show increasing heterogeneity [31].

We conducted a Bayesian NMA to examine the indirect comparison of EGFRis vs. BEV through the intermediate treatment of chemotherapy alone, and to combine the indirect comparison with the direct comparison, using WinBUGS, version 1.4.3. A detailed explanation of the statistical method [32] employed for the NMA has been provided (S2 Text). Bayesian NMAs were performed at three time points, which included: i) trials published prior to the FIRE-3 trial, [19] ii) trials up to and including FIRE-3, and iii) all trials published up to and including the CALGB 80405 trial [20], which was the last trial found by the literature search. In addition, a Bayesian NMA was conducted for all trials excluding FIRE-3. Sensitivity analyses were conducted to explore the effect of adjusting for the types of chemotherapy backbone (oxaliplatin-based, irinotecan-based or fluoropyrimidines alone) and the mode of fluoropyrimidines delivery (bolus or infusional). This was performed by including those effects as covariates in the meta-regression of the Bayesian NMA.

The results were presented according to the guidelines of the Quality of Reporting of Meta-analyses (QUOROM) and International Society for Pharmacoeconomics and Outcomes Research (ISPOR) [33,34].

## Results

### Literature Search Results

**Fig 1:**
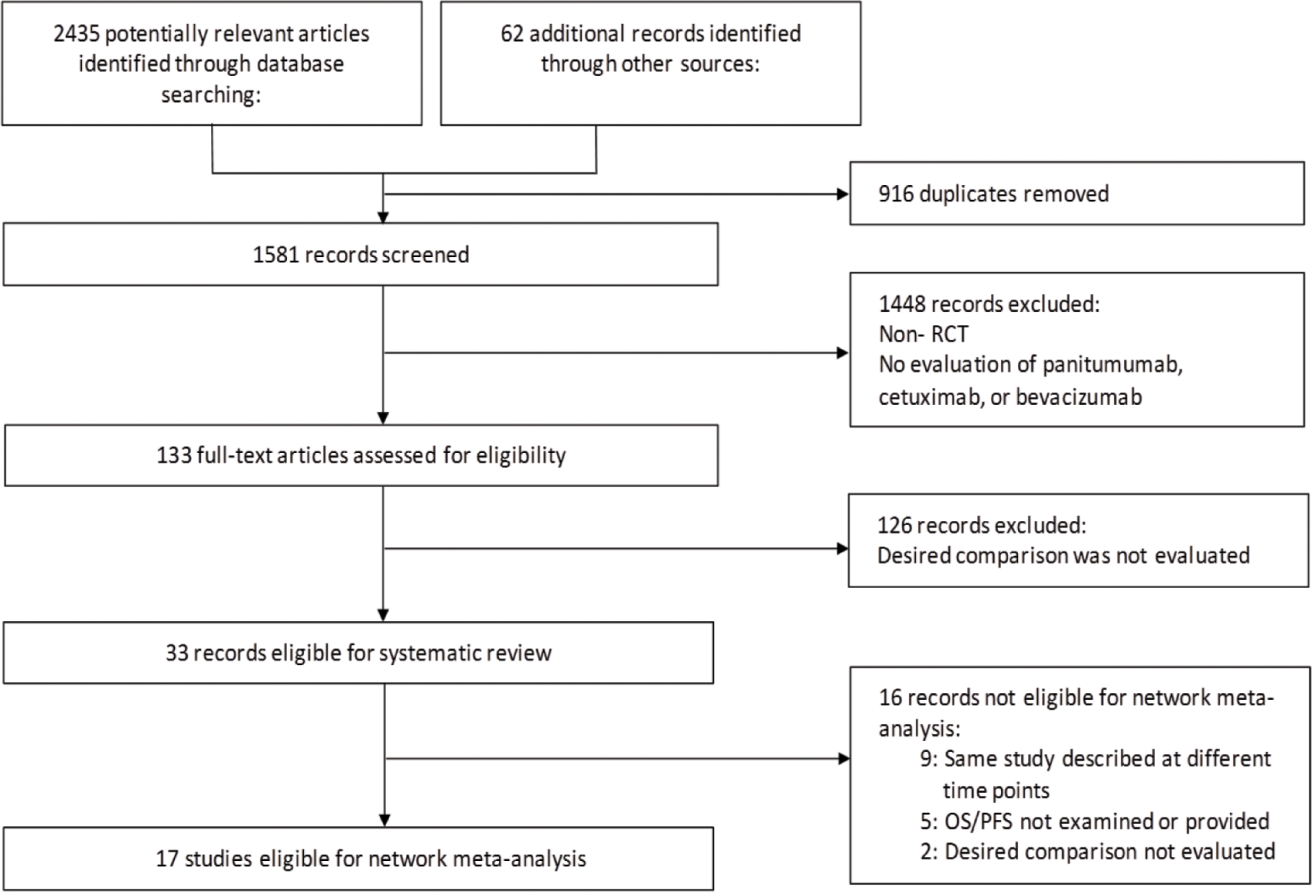
PRISMA flow diagram of included and excluded trials identified from the literature search.

**Fig 2:**
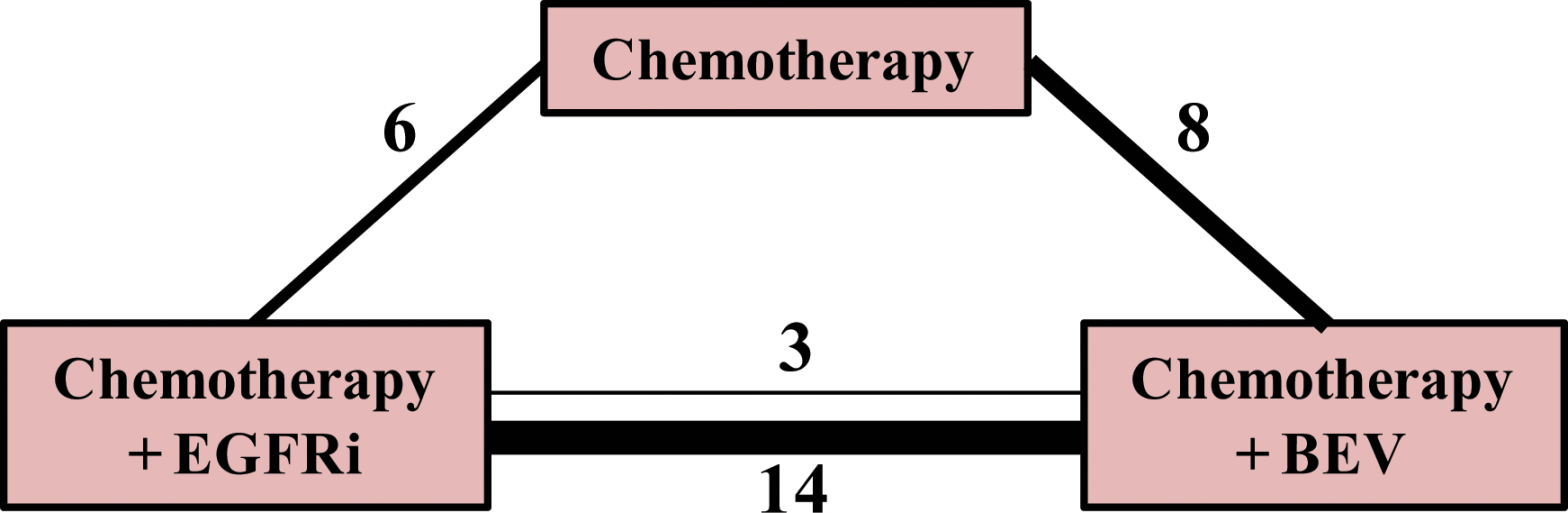
Network of treatment comparisons. The numbers represent the number of studies providing the comparison between the treatment regimens. The solid and dashed lines represent direct and indirect treatment comparisons of studies included in our analysis, respectively.

Our electronic search of Medline, Embase and the Cochrane Central Register of Controlled Trials databases yielded 2435 potentially relevant articles. Our manual search through the 2013 and 2014 ASCO General Meeting abstracts produced an additional 62 results. Following a deletion of duplicate results from different databases, there were 1581 records. Ultimately, we identified 17 unique studies for inclusion in the meta-analysis, including 2 ASCO abstracts (Fig 1). Fig 2 shows the network of available treatment comparisons, along with the number of times each comparison was made in a study.

### Study Quality

The quality of studies included in the NMA was evaluated using the Cochrane risk of bias tool [35]. A list of biases was summarized (S1 and S2 Figs). Publication bias was evaluated by constructing funnel plots for the studies included in each direct comparison for OS (S3 Fig). The three plots for OS were all symmetrical and show no evidence of publication bias. All included studies were randomized and followed intention-to-treat analysis for the primary endpoints (PFS and OS). Two of the studies (CALGB 80405, ITACA) were published only in abstract form, and we were unable to judge whether selection, allocation, assessment, attrition and/or reporting bias were present. All of the trials reported median PFS, and all except for one (Kabbinavar et al, 2003), provided extractable data for OS. Nine of the studies did not blind the patients and assessors, leading to a possible assessment bias with regards to our primary endpoint, PFS. Heterogeneity was present in some pairwise treatment comparisons based on I^2^; however, the studies were comparable in terms of patient characteristics and outcomes.

### Trial Characteristics

All included studies were randomized and specific for the first-line treatment of metastatic colorectal cancer. Table 1 summarizes the baseline characteristics of the included trials. Each trial had a chemotherapy regimen in both arms, and either BEV or EGFRi in at least one arm of the trial. In total, BEV plus chemotherapy was compared to chemotherapy alone in eight studies, an EGFRi plus chemotherapy was compared to chemotherapy alone in six studies, and BEV was compared to an EGFRi—with chemotherapy administered in both arms- in three studies. All trials included in the meta-analysis reported median PFS and OS.

**Table 1:** Characteristics of eligible trials. The demographics included in the above table pertain only to patients included in our analysis.

	Year	Chemotherapy Backbone	Treatment	Number of Patients enrolled	Median Age (years)	ECOG status(%)	Follow up (months)	PFS (months)	OS (months)
**Studies comparing BEV + chemo vs. chemo alone**									
**Guan (ARTIST)** ^**1**^	2011	Irinotecan, Folinic acid, Fluorouracil^a^	Chemo	64	50	0 = 36, 1 = 64	Every 3 months until death.	4.2	13.4
			BEV + chemo	139	53	0 = 47, 1 = 53		8.3	18.7
**Hurwitz** ^**1**^	2004	Irinotecan,Folinic acid, Fluorouracil^a^	Chemo	411	59	0 = 55, 1 = 44, 2 = <1	Until death, loss to follow-up, or termination of the study	6.2	15.6
			BEV + chemo	402	60	0 = 58, 1 = 41, 2 = <1		10.6	20.3
**Kabbinavar** ^**1**^	2003	Folinic acid, Fluorouracil^a^	Chemo	36	Not available	0 = 61,1 = 39	2 months until death or loss to follow-up	5.2	13.8
			BEV + chemo	35		0 = 60, 1 = 40		9.0	21.5
**Kabbinavar (AVF2192)** ^**1**^	2005	Folinic acid, Fluorouracil^a^	Chemo	105	71	0 = 28, 1 = 67, 2 = 6	Every 4 months until death, loss to follow-up, or termination of the study	5.5	12.9
			BEV + chemo	104	71	0 = 29, 1 = 64, 2 = 8		9.2	16.6
**Passardi (ITACA)** ^**1**^	2013	Oxaliplatin, Folinic acid, Fluorouracil^a^,^b^ *OR* Irinotecan, Folinic acid, Fluorouracil^a^,^b^	Chemo	194	Not available	Not available	18.4	8.4	20.6
			BEV + chemo	176				9.2	20.6
**Saltz (NO16966)** ^**1**^	2008	Oxaliplatin, Capecitabine *OR* Oxaliplatin, Folinic acid, Fluorouracil^b^	Chemo	701	60	0 = 60, 1 = 40,	27.6	8.0	19.9
			BEV + chemo	699	60	0 = 58, 1 = 42, 2 = <1		9.4	21.3
**Saunders (AVEX)** ^**2**^	2013	Capecitabine	Chemo	140	76	Not available	Not available	5.1	16.8
			BEV + chemo	140	76			9.1	20.7
**Tebbutt (MAX)** ^**2**^	2010	Capecitabine	Chemo	156	69	0 = 58, 1 = 38, 2 = 4	30.8	5.7	18.9
			BEV + chemo	157	67	0 = 58, 1 = 34, 2 = 8		8.5	18.9
**Studies comparing EGFRis + chemo vs. chemo alone**									
**Bokemeyer (OPUS)** ^**3**^	2011	Oxaliplatin, Folinic acid, Fluorouracil^a^,^b^	Chemo	97	60	0 = 39, 1 = 51, 2 = 10	Not available	7.2	18.5
			EGFRi + chemo	82	62	0 = 39, 1 = 54, 2 = 7		8.3	22.8
**Douillard (PRIME)** ^**4**^	2010	Oxaliplatin, Folinic acid, Fluorouracil^a^,^b^	Chemo	331	62	0–1 = 94, ≥2 = 6	12.5	8.0	19.7
			EGFRi + chemo	325	62	0–1 = 94 ≥2 = 6	13.2	9.6	23.9
**Maughan (COIN)** ^**3**^	2011	Oxaliplatin, Capecitabine, OR Oxaliplatin, Folinic acid, Fluorouracil^a^,^b^	Chemo	367	63	0 = 48, 1 = 45, 2 = 7 **WHO performance status	21	8.6	17.9
			EGFRi + chemo	362	63	0 = 47, 1 = 47, 2 = 6	23	8.6	17.0
**Tveit (NORDIC-VII)** ^**3**^	2012	Oxaliplatin, Folinic acid, Fluorouracil^a^	Chemo	97	60	0 = 73, 1 = 22, 2 = 5 **WHO performance status	Not available	8.7	22.0
			EGFRi + chemo	97	60	0 = 68, 1 = 28, 2 = 4		7.9	20.1
**Van Cutsem (CRYSTAL)** ^**3**^	2011	Irinotecan, Folinic acid, Fluorouracil^a^,^b^	Chemo	350	59	0 = 57, 1 = 39, 2 = 4	29.4	8.4	20.0
			EGFRi + chemo	316	61	0 = 58, 1 = 38, 2 = 4	29.9	9.9	23.5
**Ye** ^**3**^	2013	Oxaliplatin, Folinic acid, Fluorouracil^a^,^b^ *OR* Irinotecan, Folinic acid, Fluorouracil^a^,^b^	Chemo	68	59	0 = 79, 1 = 21	25.0	5.8	21.0
			EGFRi + chemo	70	57	0 = 83, 1 = 17	25.0	10.2	30.9
**Studies comparing BEV + chemo vs. EGFRis + chemo**									
**Heinemann (FIRE-3)** ^**1**^,^**3**^	2013	Irinotecan, Folinic acid, Fluorouracil^a^,^b^	EGFRi + chemo	297	Not available	0 = 52,1 = 46, 2 = 2	Every 3 months until death, for a maximum of 5 years	10.0	28.7
			BEV + chemo	295		0 = 54,1 = 45, 2 = 1		10.3	25
**Shwartzberg(PEAK)**	2014	Oxaliplatin, Folinic acid, Fluorouracil^a^,^b^	EGFRi + chemo	142	63	0 = 63, 1 = 37	Not available	10.9	34.2
			BEV + chemo	143	61	0 = 64, 1 = 36		10.1	24.3
**Venook (CALGB 80405)** ^**1**^,^**3**^	2014	Oxaliplatin, Folinic acid, Fluorouracil^a^,^b^ *OR* Irinotecan, Folinic acid, Fluorouracil^a^,^b^	EGFRi + chemo	578	59	Not available	24	10.4	29.9
			BEV + chemo	559	59			10.8	29.0

^a^ Given as a bolus

^b^ Given as an infusion

^1^ Dose of BEV was 5 mg/kg

^2^ Dose of BEV was 7.5 mg/kg

^3^ Dose of CET was 400 mg/m^2^ initial dose followed by 250 mg/m^2^ the following week

^4^ Does of PAN was 6 mg/kg every 2 weeks

All of the included studies were comparable in terms of patient characteristics. The PFS of the chemotherapy-only reference arms ranged between 5.2–8.7 months, while the OS of these reference arms ranged between 13.8–22.0 months. Median PFS and OS were lower in the chemotherapy reference arm in trials examining the efficacy of BEV than in trials examining an EGFRi. Of the fourteen trials that compared an EGFRi or BEV with chemotherapy alone, eleven found a statistically significant difference in PFS, and five found a statistically significant difference in OS. Three studies comparing BEV and EGFRis directly did not find statistically significant differences in PFS, and one study found a difference in OS.

### Comparison of regimens: Pairwise direct meta-analyses

**Fig 3:**
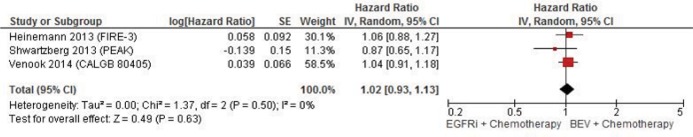
Forest plots of hazard ratios comparing progression-free survival of EGFRis with chemotherapy versus BEV with chemotherapy.

**Fig 4:**
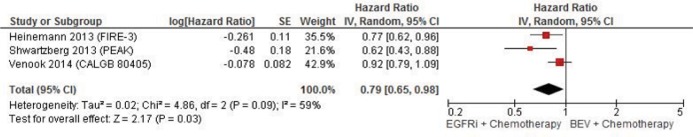
Forest plots of hazard ratios comparing overall survival of EGFRis with chemotherapy versus BEV with chemotherapy.

Pairwise comparisons of trials examining the efficacy of the same antibody therapy were made first using a random-effects model. Direct pairwise meta-analyses comparing EGFRis versus BEV with chemotherapy in both arms did not detect a difference between the two arms with respect to PFS, HR = 1.02 (CI: 0.93–1.13). However, with respect to OS, a statistically significant difference was seen in favor of the EGFRis arm, HR = 0.79 (CI: 0.65–0.98). The results of PFS and OS comparisons are shown in Figs 3 and 4, respectively. Forest plots of the hazard ratios for PFS and OS between EGFRis vs. chemotherapy alone, and between BEV and chemotherapy alone are available (S4 and S5 Figs, respectively).

### Indirect and network meta-analyses

**Fig 5:**
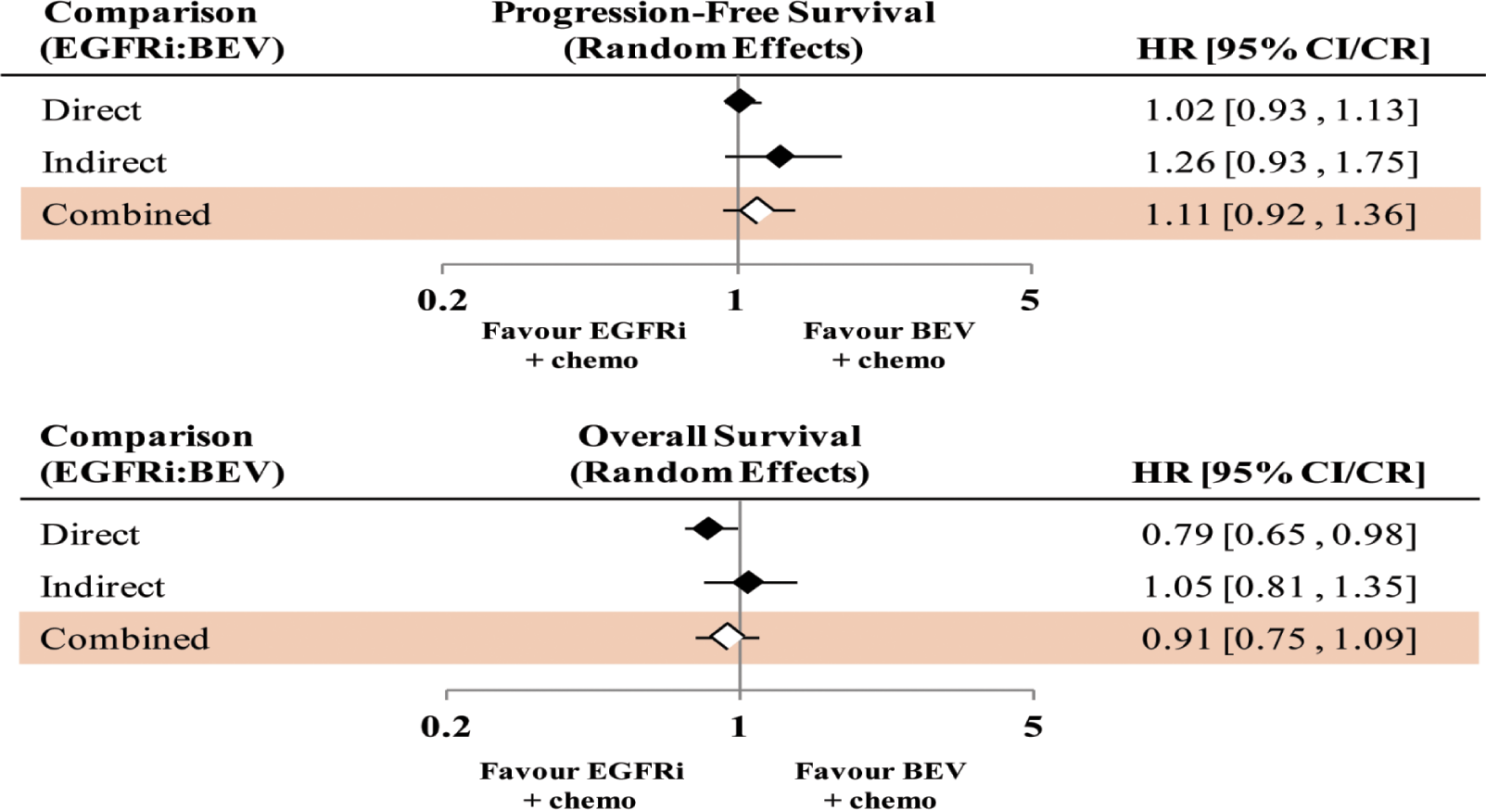
Forest plots showing hazard ratios calculated from direct, indirect and combined analysis of EGFRis versus BEV regimens. For direct comparisons a CI was calculated, and for indirect and combined comparisons, a CR was calculated.

**Fig 6:**
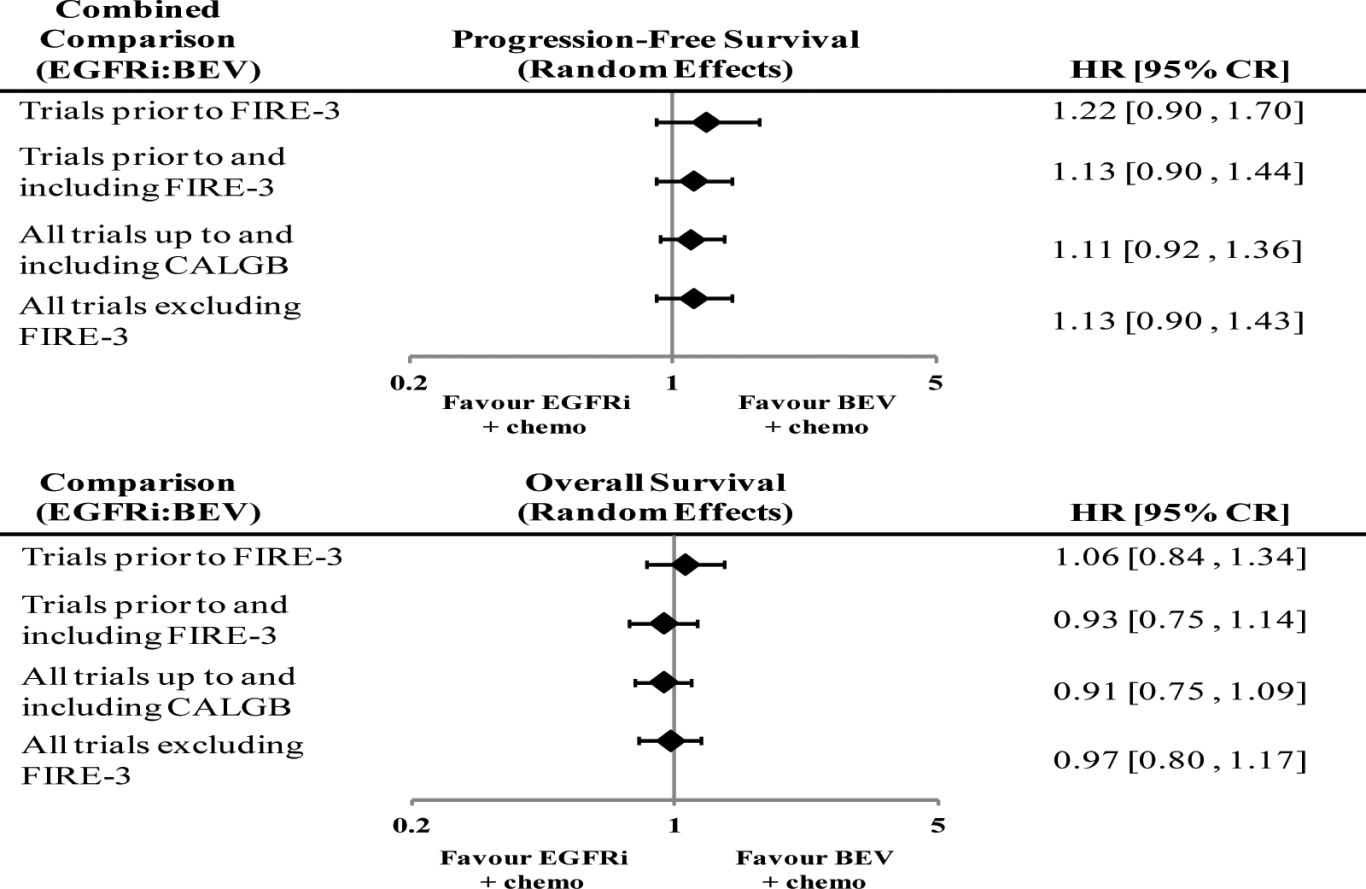
Forest plots showing hazard ratios for calculated from combined comparison of: trials prior to FIRE-3, trials up to and including FIRE-3, all trials up to and including CALGB, all trials excluding FIRE-3.

Indirect comparisons of EGFRis versus BEV (through the intermediate of chemotherapy only: 6 RCTs comparing EGFRis and chemotherapy versus chemotherapy only, 8 RCTs comparing BEV and chemotherapy vs. chemotherapy alone) showed that PFS HR = 1.26 (95% CR: 0.93–1.75) and OS HR = 1.05 (95% CR: 0.81–1.35). Combining the direct and indirect comparisons (17 RCTs) showed a PFS HR = 1.11 (95% CR: 0.92–1.36) in favor of BEV therapy, while OS was in favor of EGFRi therapy, HR = 0.91 (95% CR: 0.75–1.09), although neither result was statistically significant. Fig 5 shows the results of direct pairwise meta-analysis, indirect comparison, and combined analysis for the comparison of EGFRis with BEV. A summary of these results has been provided (S6 Fig). Fig 6 shows a comparison of HRs for combined comparison of: trials prior to FIRE-3, trials up to and including FIRE-3, all trials up to an including CALGB 80405, and all trials excluding FIRE-3. The successive inclusions of FIRE-3 and CALGB trials over time did not change the results that neither EGFRis nor BEV was superior to the other statistically, with increasing precisions with more trials included.

### Sensitivity Analysis

The results of sensitivity analyses adjusting for the effect of the types of chemotherapy backbone and mode of fluoropyrimidine delivery (bolus vs. infusional) have been provided (S7 Fig). The findings are the same as the main base case results of the network meta-analyses for OS and PFS.

## Discussion

We conducted a systematic review and NMA of randomized controlled trials to examine whether EGFRis or BEV is associated with improved PFS and OS in the first-line treatment of patients with mCRC. The trials included in our study compared either EGFRis with chemotherapy vs. chemotherapy alone, BEV with chemotherapy vs. chemotherapy alone, or EGFRis vs. BEV with chemotherapy in both arms. Pairwise meta-analyses were conducted to compare direct evidence, and an NMA was conducted using the intermediate treatment of chemotherapy for an indirect comparison. The direct and indirect evidence was combined to determine which therapy better improves survival outcomes.

The results of our NMA did not show a benefit to OS with EGFRi therapy, a trend which was also seen by the indirect comparison of treatment regimens. However, results from direct meta-analysis showed an improvement in survival with EGFRi therapy. This may be due to the large weight of the FIRE-3 trial (592 patients) on our direct analysis, which found a statistically significant improvement in OS with EGFRi therapy (HR = 0.77, p = 0.017). The results of the larger direct comparison CALGB 80405 trial (1137 patients)—the only one powered for OS—were congruent with our NMA results. Examining the OS HR with and without the inclusion of FIRE-3 results demonstrates the impact of the FIRE-3 trial on our combined results (HR = 0.91 (95% CR: 0.75–1.09) and HR = 0.94 (95% CR: 0.76–1.15)), respectively. In the FIRE-3 trial, the separation of the Kaplan-Meyer OS curves only after 18 months of treatment, which combined with the lack of differences in the PFS, suggests the improvement in the OS may be related to post-progression events. This phenomenon, which FIRE-3 investigators suggested may be related to the increased depth of response to EGFRi therapy, was not observed in other trials comparing EGFRi vs. chemotherapy alone [36], CALGB 80405, our indirect comparisons of EGFRis vs. BEV, or our combined NMAs. The discrepancy about the OS endpoint between the combined NMA and the direct comparison, influenced by FIRE-3, may suggest that FIRE-3 is an outlier statistically. The PFS results from our NMA were concordant with results from our direct pairwise meta-analysis, showing no improvement of PFS with EGFRi therapy. The outcomes in all other trials (including PFS in the FIRE-3 trial) did not show a benefit with EGFRis.

Two separate sensitivity analyses were conducted to adjust for the possible confounding effect of 1) chemotherapy backbone and 2) mode of fluoropyrimidine delivery (bolus vs. infusional) on our results. PFS and OS have been shown in the literature [37,38]—as well as in the control arms of trials included in our study (Table 1)—to vary with the chemotherapy regimen used, and were adjusted for. Similarly, we adjusted for the potential confounder of fluoropyrimidine delivery mode (bolus vs. infusional) [39]. Our sensitivity analyses showed similar results between each sensitivity analysis, and the unadjusted NMA, for both PFS and OS, suggesting the robustness of our results.

The results of our pairwise meta-analyses showed an improvement in survival with the addition of EGFRis or BEV to chemotherapy, which is consistent with similar studies in the literature and previous pairwise meta-analyses [40, 41, 42,43]. The heterogeneity in our study may be due to the different chemotherapy backbones used in the studies, as variation in survival outcomes was also observed in the chemotherapy reference arms. Furthermore, even though the funnel plots were symmetrical and did not suggest evidence of publication bias, their ability to detect publication bias was less sensitive due to the relatively small number of trials in each forest plot [44]. The EGFRi pairwise meta-analysis also included studies with different biologics added (cetuximab and panitumumab), which may have contributed to heterogeneity. Studies included in the NMA were comparable in terms of patient characteristics.

In our analysis, we examine K-RAS wild type tumors because K-RAS was previously recognized and accepted as the biomarker of choice for selecting patients for EGFR inhibitors prior to 2014. Recently, pan RAS (as known as extended RAS) has been recognized as the biomarker of choice for selecting patients for EGFRis since 2014 [45]. Therefore, in future analyses, it would be important to look at how the BEV vs. EGFRi therapies compare in terms of outcomes with pan RAS wide type (i.e. also with exon 3 and NRAS wild-type patients) [44]. Updated data from FIRE-3, PEAK, and CALGB 80405 trials will also provide further evidence to refine these findings.

## Conclusion

Our NMA reviewed and analyzed the existing literature for RCTs examining EGFRi and BEV treatments for metastatic colorectal cancer in the first-line setting. The results of our NMA did not show a statistical difference between EGFRis and BEV therapies with regards to both PFS and OS. The results of the NMA were congruent with indirect analysis with respect to both PFS and OS, as well as with direct analysis with respect to PFS. The findings of CALGB appeared to be congruent with the collective synthesis of the existing literature, while the findings of FIRE-3 appeared to be incongruent with the remaining literature. Further evidence from ongoing trials, which directly compare EGFRis and BEV therapies, will further validate of our results.

## Supporting Information

S1 Fig Risk of bias summary for included trials organized by domain. (TIF)

S2 Fig Risk of bias summary for included trials organized by study. (TIF)

S3 Fig Publication bias assessments of overall survival for trials. a) BEV + Chemotherapy vs. Chemotherapy alone, b) EGFRis + Chemotherapy vs. Chemotherapy alone and c) EGFRis + Chemotherapy vs. BEV + Chemotherapy
(TIF)

S4 Fig Forest plots of hazard ratios for progression-free survival comparing a) EGFRis with chemotherapy versus chemotherapy alone and b) BEV with chemotherapy versus chemotherapy alone. (TIF)

S5 Fig Forest plots of hazard ratios for overall survival comparing a) EGFRis with chemotherapy versus chemotherapy alone and b) BEV with chemotherapy versus chemotherapy alone. (TIF)

S6 Fig Summary of hazard ratios with credible regions for direct comparisons between treatment regimens for a) progression-free survival and b) overall survival. (TIF)

S7 Fig Forest plots showing hazard ratios for progression-free survival and overall survival calculated for non-adjusted combined analysis of EGFRis vs. BEV treatment regimens, chemotherapy backbone adjusted HRs, and fluoropyrimidine delivery mode adjusted HRs. (TIF)

S1 PRISMA Checklist PRISMA checklist for reporting our systematic review and meta-analysis. (PDF)

S1 Text Search strategy used to identify trials. (DOCX)

S2 Text Statistical analysis method used to create the Bayesian MTC. (DOCX)
