# Supplementary figures and images for: A Systematic Review and Network Meta-Analysis of Biologic Agents in the First Line Setting for Advanced Colorectal Cancer
Source: PLoS One. 2015 Oct 16;10(10):e0140187. doi: 10.1371/journal.pone.0140187 (PMC4608731; doi:10.1371/journal.pone.0140187)

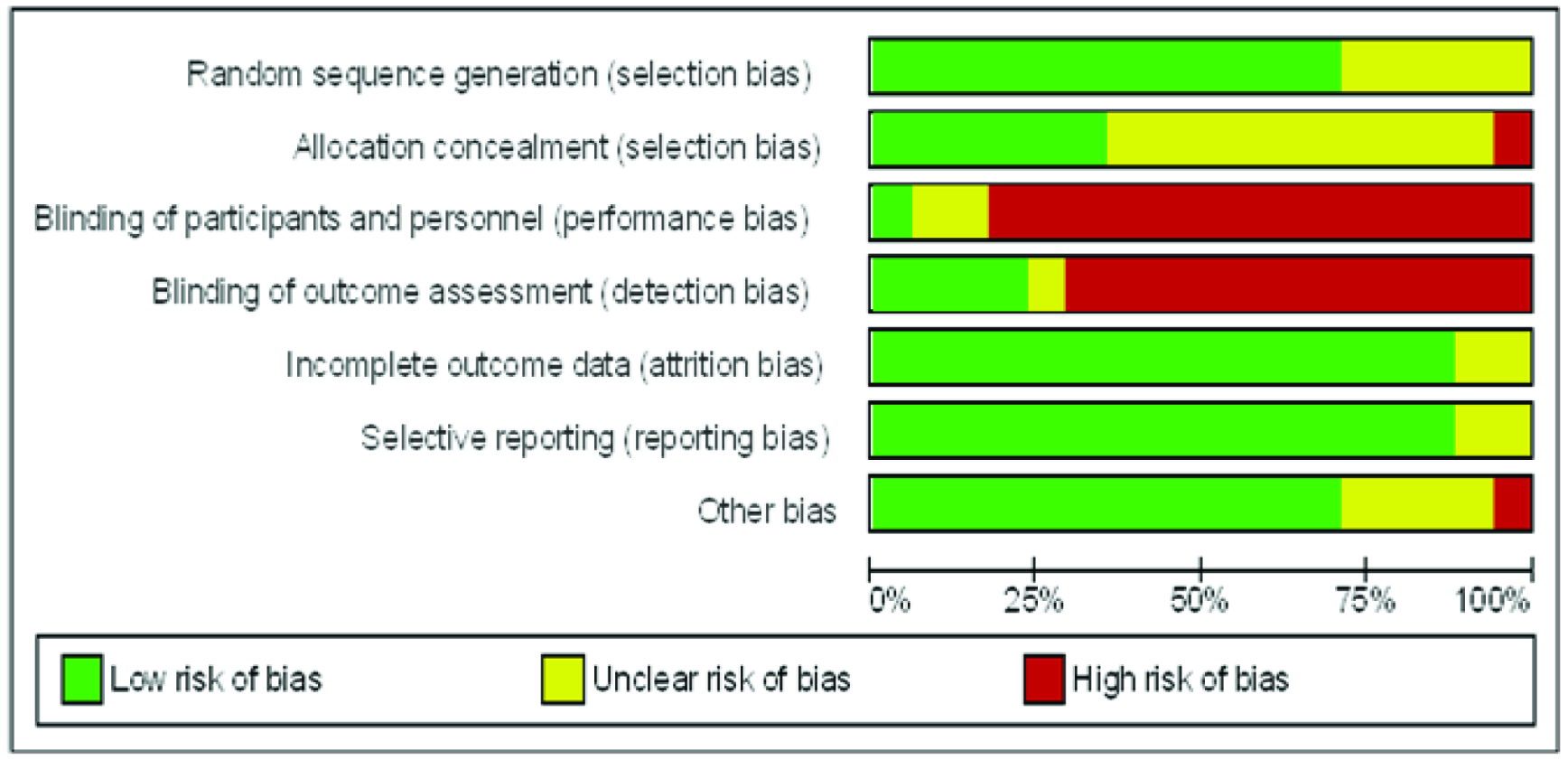

Supplement: S1 Fig — (TIF) [file pone.0140187.s001.tif]

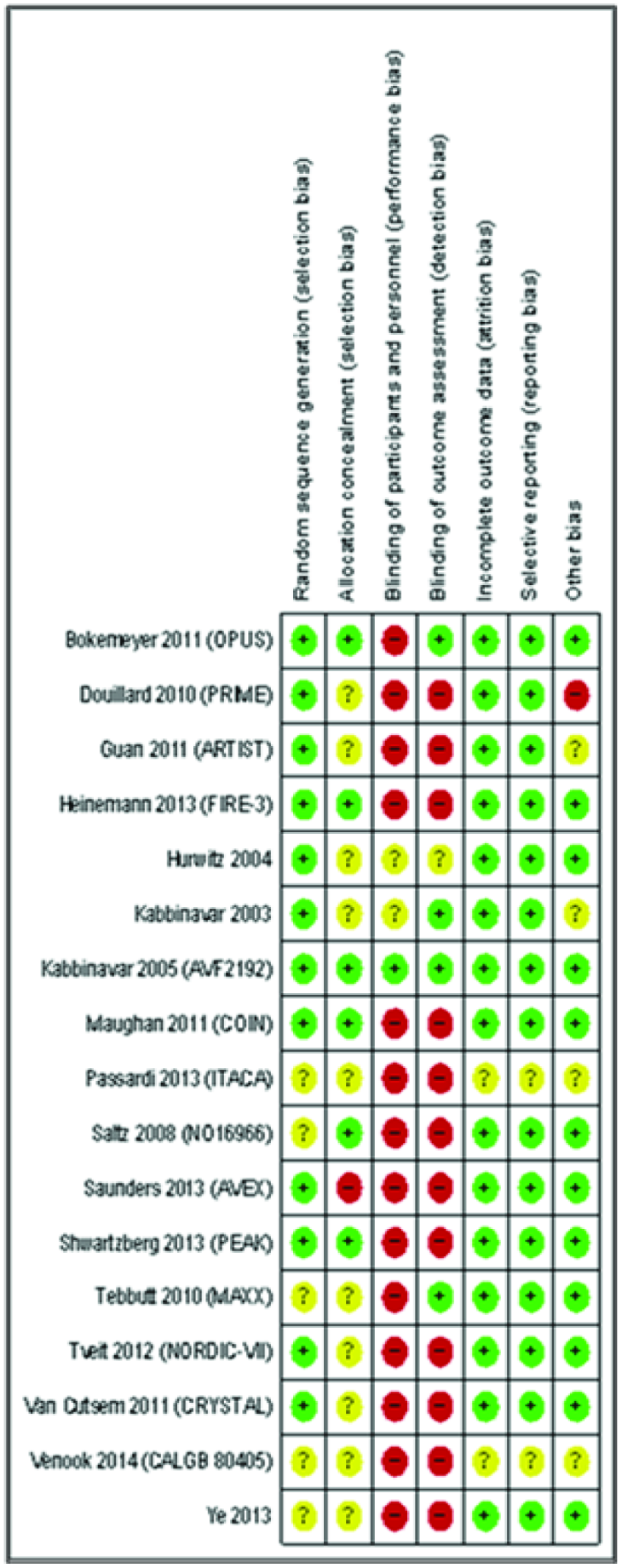

Supplement: S2 Fig — (TIF) [file pone.0140187.s002.tif]

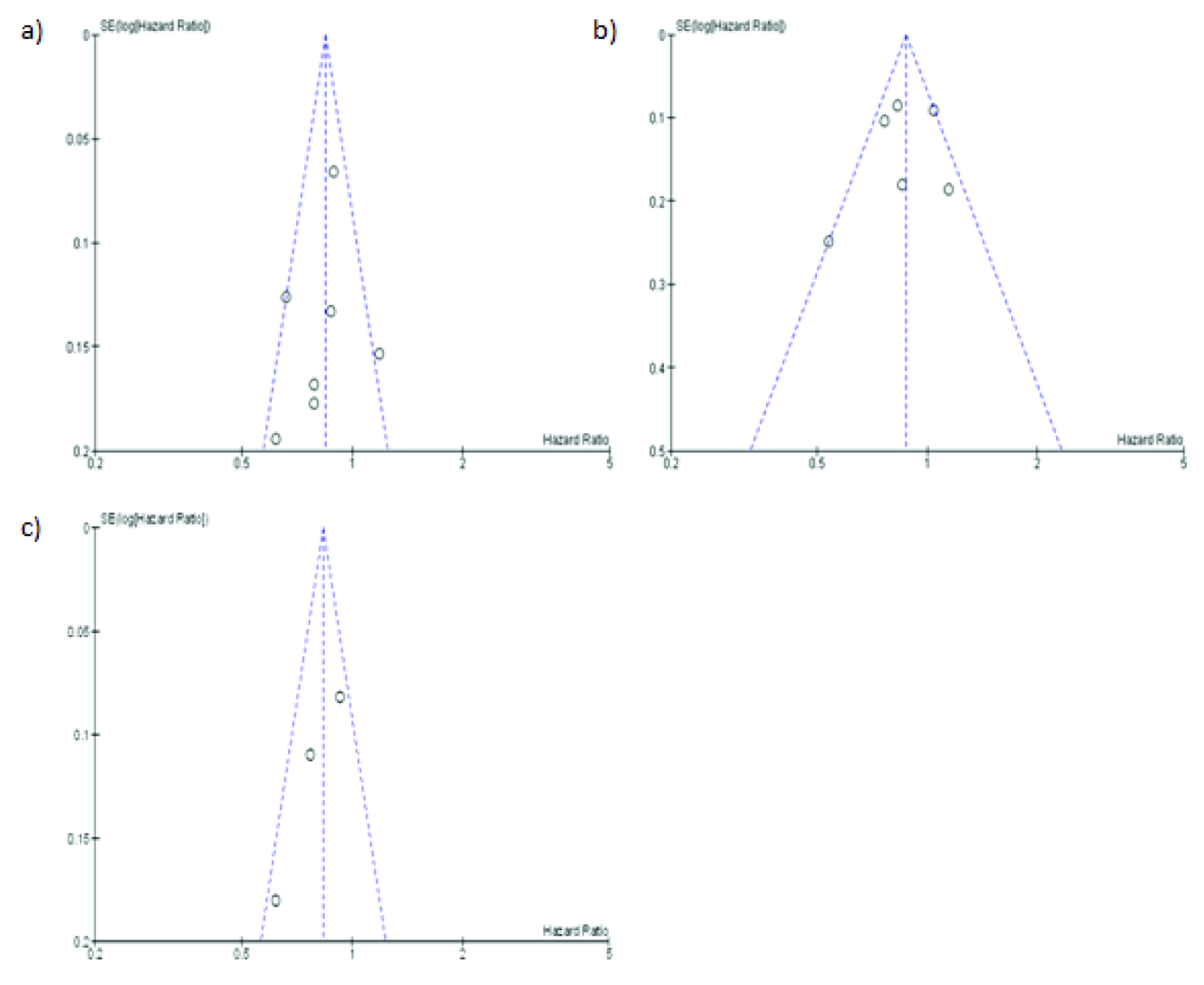

Supplement: S3 Fig — a) BEV + Chemotherapy vs. Chemotherapy alone, b) EGFRis + Chemotherapy vs. Chemotherapy alone and c) EGFRis + Chemotherapy vs. BEV + Chemotherapy (TIF) [file pone.0140187.s003.tif]

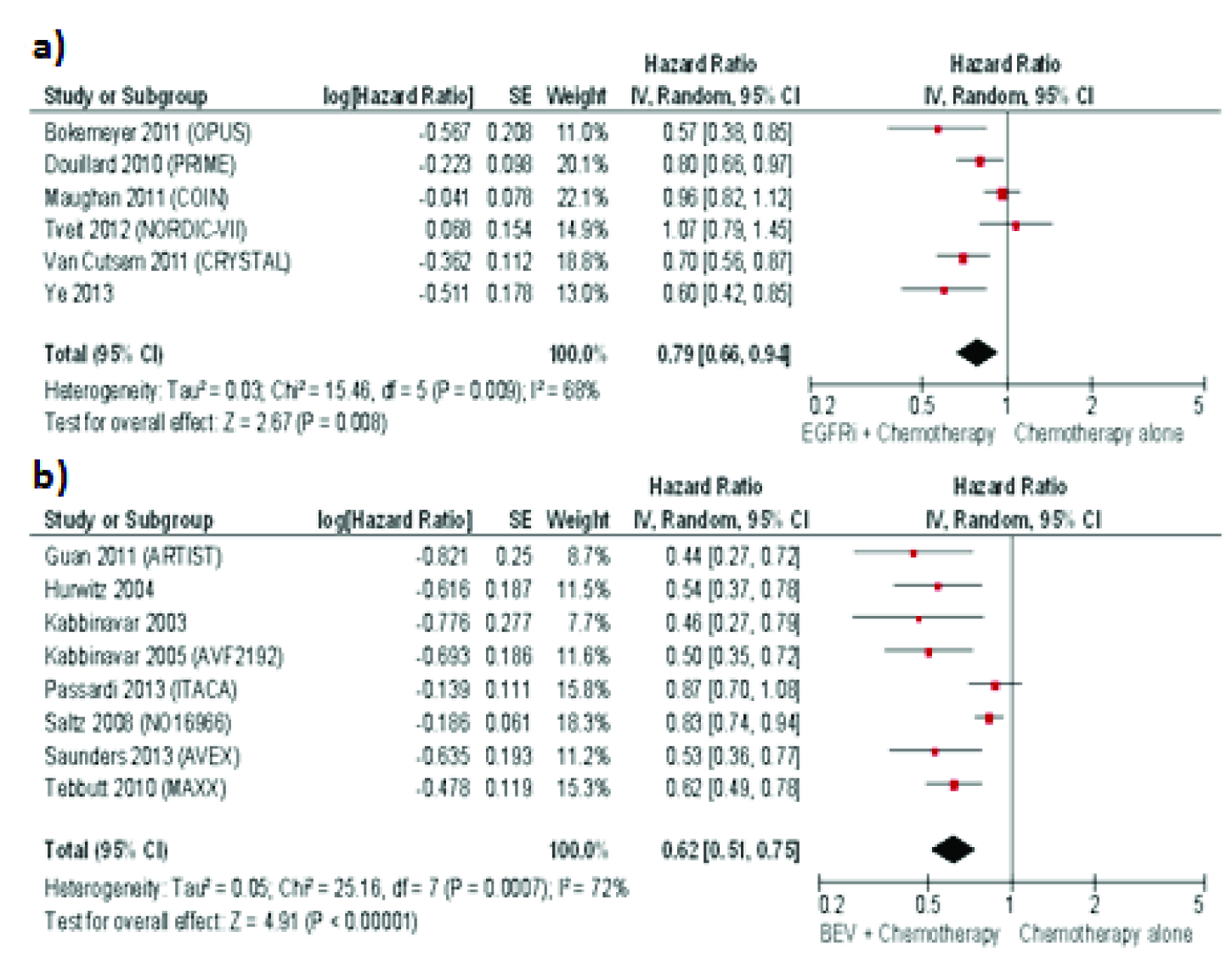

Supplement: S4 Fig — (TIF) [file pone.0140187.s004.tif]

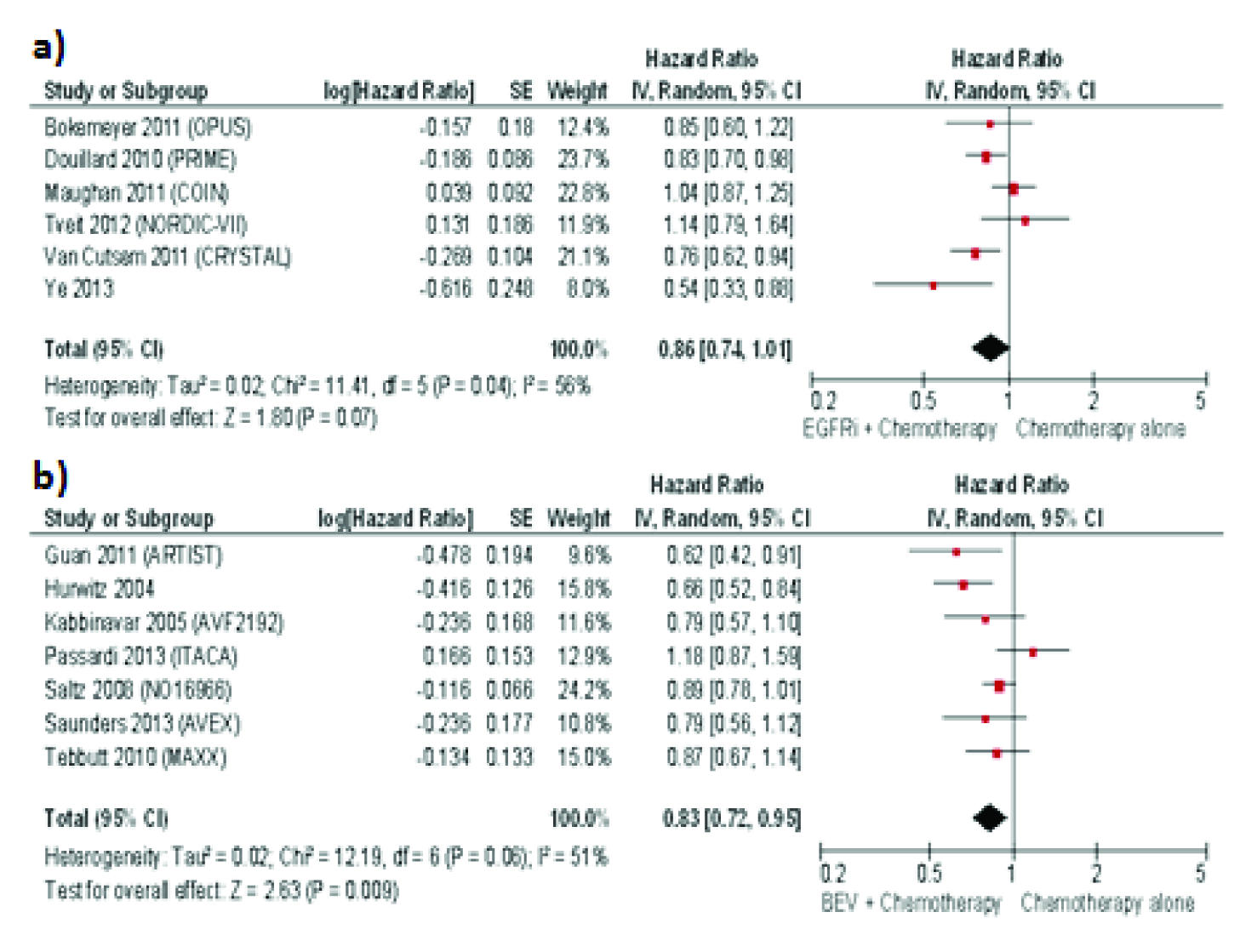

Supplement: S5 Fig — (TIF) [file pone.0140187.s005.tif]

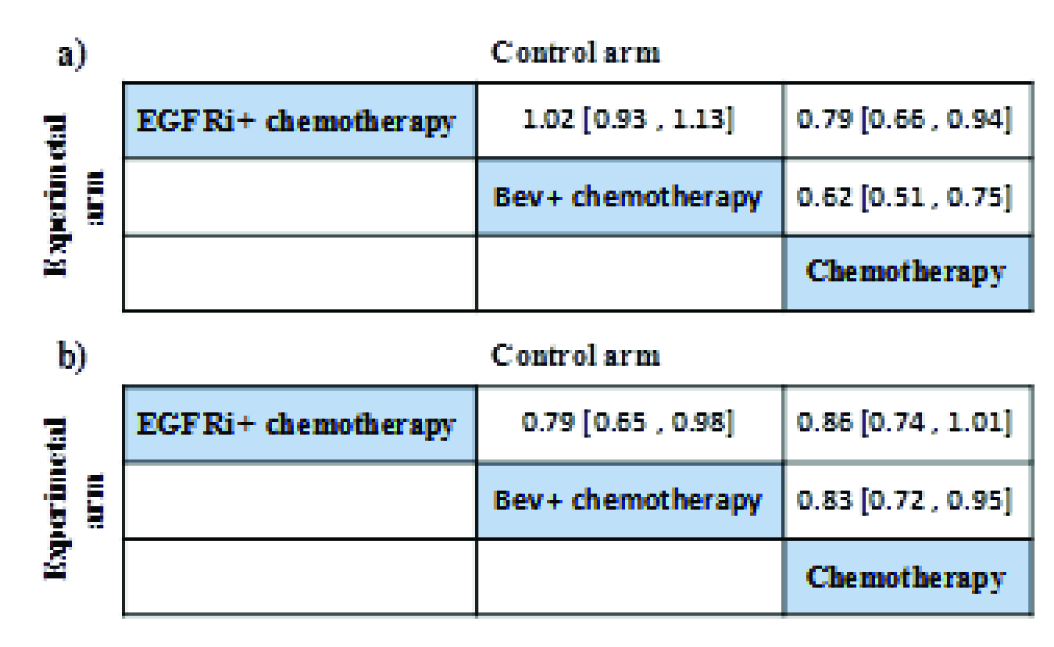

Supplement: S6 Fig — (TIF) [file pone.0140187.s006.tif]

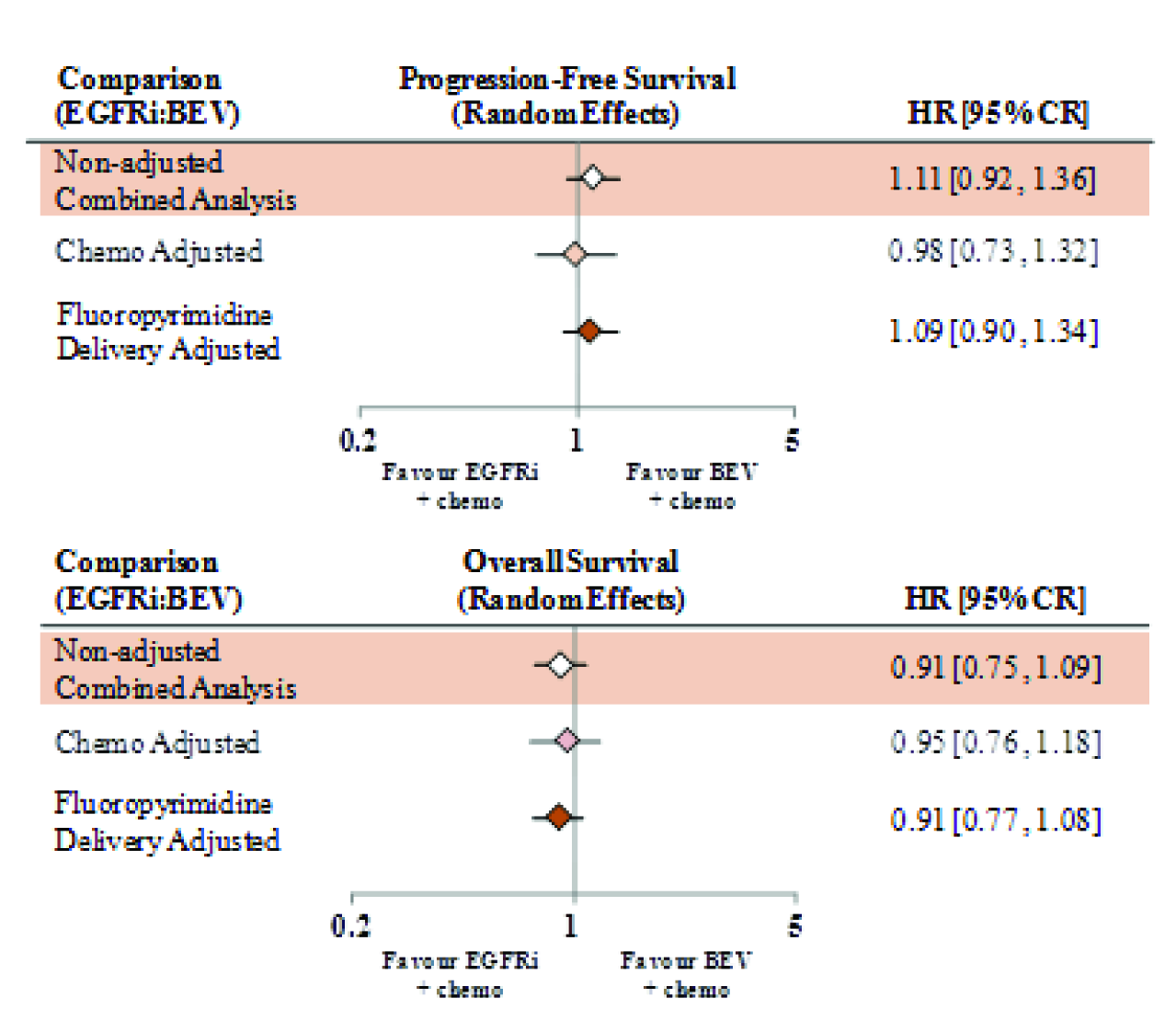

Supplement: S7 Fig — (TIF) [file pone.0140187.s007.tif]
