# Supplementary material for: A Systematic Review and Network Meta-Analysis of Biologic Agents in the First Line Setting for Advanced Colorectal Cancer
Source: PLoS One. 2015 Oct 16;10(10):e0140187. doi: 10.1371/journal.pone.0140187 (PMC4608731; doi:10.1371/journal.pone.0140187)
Supplement: S1 Text — (DOCX) [file pone.0140187.s009.docx]

Search strategy used the following limits alone, or in combination: 1) terms describing cancer (i.e., “cancer” and “neoplasm”); 2) terms describing colorectal (“colorectal”, “colon”, “rectal”, “rectum”, or “sigmoid”); 4) chemotherapeutics (ie, “irinotecan”, “camptosar”, “cpt-11 or cpt11”, “oxaliplatin”, “eloxatin”, “FOLFOX”, “XELOX”, “CAPEOX”, “FOLFIRI”, “XELIRI”, “CAPIRI”, or “CAPOX”); 5) monoclonal antibody therapies (ie, “bevacizumab”, “avastin”, “cetuximab”, “erbitux”, “panitumumab”, “vectibix”); 6) randomized trials (ie, “random”, “blind”, “mask”, “RCT”, or “placebo”). In addition, we manually searched through abstracts submitted to the 2013 and 2014 American Society of Clinical Oncologists (ASCO) general meeting for applicable trials.
