# Supplementary material for: A Systematic Review and Network Meta-Analysis of Biologic Agents in the First Line Setting for Advanced Colorectal Cancer
Source: PLoS One. 2015 Oct 16;10(10):e0140187. doi: 10.1371/journal.pone.0140187 (PMC4608731; doi:10.1371/journal.pone.0140187)
Supplement: S2 Text — (DOCX) [file pone.0140187.s010.docx]

Using WinBUGS, 3 chains were fit with 40,000 burn-ins and 40,000 iterations each. We used the following non-informative prior distributions: uniform (0, 2) for standard deviation of the random effects model and normal (0, tau=0.0001) for log[HR]s. Non-informative priors were used because this allowed the trial data to inform the results, rather than letting strong priors dictate the results. Effect sizes for the Bayesian network meta-analysis were described with 95% credible regions (CRs), since “credible” is a more appropriate term than “confidence” when conducting Bayesian mixed treatment comparisons in which direct and indirect evidence are combined. An assessment of convergence was done using model diagnostics, such as trace plots and the Brooks-Gelman-Rubin statistic.^1^ The most appropriate fit for the model was determined based on the residual deviance and deviance information criterion (DIC) for each of the measured outcomes.

^1^ Ntzoufras I. Bayesian modeling using WinBUGS. New York: Wiley; 2009.
